# An Ex Vivo Patient-Derived Tumor-Bearing Human Kidney Model Recapitulates Drug Toxicity and Metabolic Distribution

**DOI:** 10.34133/research.1257

**Published:** 2026-05-21

**Authors:** Yaowei Li, Zhe Wang, Qi Liu, Ziyi Liu, Haonan Li, Haiqiang Duan, Shiyu Huang, Qing Shi, Yubo Zhao, Jiaqian Xu, Tianxi Yu, Guangzheng Wu, Hongjian Song, Changhao Zhao, Yishuo Yan, Zhishuai Zhang, Zongzheng Yang, Zhihao Yin, Jianwei Wang, Peng Zhang, Peng Dai, Dayong Hou, Zhichao Tong, Ziqi Wang

**Affiliations:** ^1^ NHC Key Laboratory of Molecular Probe and Targeted Theranostics, Harbin Medical University Cancer Hospital, Harbin Medical University, Harbin 150001, China.; ^2^ Department of Urology, Harbin Medical University Cancer Hospital, Harbin 150001, China.; ^3^ Department of Urology, Second Affiliate Hospital of Harbin Medical University, Harbin 150001, China.; ^4^ Department of Cancer System Imaging, The University of Texas MD Anderson Cancer Center, Houston, TX, USA.; ^5^ Heilongjiang Provincial Key Laboratory of Basic Medical Sciences in Urology Cancer, Harbin Medical University Cancer Hospital, Harbin 150001, China.; ^6^ Biobank, Harbin Medical University Cancer Hospital, Harbin 150001, China.; ^7^ Department of Urogenital Medical Oncology, Harbin Medical University Cancer Hospital, Harbin 150001, China.; ^8^ Department of Cystoscope Center, Harbin Medical University Cancer Hospital, Harbin 150001, China.

## Abstract

Renal metabolic behavior constitutes a critical evaluation parameter during drug development, toxicity assessment, and biodistribution. However, the inherent biological architecture of existing animal and cell-based models fails to recapitulate the authentic metabolic processes and spatial distribution patterns of drugs in the human kidney at a macroscopic level. Therefore, developing a standardized human-derived kidney model was necessary. In this work, the ex vivo tumor-bearing kidney (ETK) model was established using kidneys from 50 clinically diagnosed patients after the radical surgery. The standardized ETK model included the following: (a) selected according to the proportion of preserved normal tissue, vascular status suitable for ETK perfusion, and overall structural integrity of the kidneys; (b) surgical preparation exposed the renal pelvis and enabled catheterization of the renal artery and ureter; (c) perfused with hypothermic (4 to 10 °C) saline at a flow rate of 70 ml/min within perfusion system; and (d) ETK detection efficiency and drug biodistribution were assessed using biological sample indicators. Results showed that ETK maintained intact glomerular and tubular microstructures throughout perfusion and produced stable urine output with preserved filtration capacity. When treated with different kinds of nephrotoxic agents (cisplatin, gentamicin, and cephalosporins), the ETK model reproduced drug-specific injury patterns and exhibited biochemical changes and histological features that were consistent with real clinical observations. In drug-distribution analysis, indocyanine green (ICG) showed a negative fluorescence tumor contrast and enriched fluorescence in the normal kidney in the ETK model, which were consistent with real clinical observations, whereas mouse orthotopic renal cancer models showed positive ICG tumor accumulation, indicating species-dependent differences in renal drug distribution. Overall, the ETK model provided a structurally preserved and functionally responsive human-derived renal system for investigating renal drug metabolism, nephrotoxicity, tumor imaging, and drug-delivery strategies.

## Introduction

The human kidney serves not only as a principal organ for the elimination of metabolic waste and toxic compounds but also as a central site for drug disposition [1]. Approximately 30% to 40% of clinically used drugs rely primarily on glomerular filtration, active tubular secretion, or tubular reabsorption for clearance [2]. These drugs and their metabolites traversed the high-throughput circulation of the renal artery [3] and were processed within nearly one million nephrons [4]. Along each nephron, a broad repertoire of transporters—such as organic anion transporters (OATs), organic cation transporters (OCTs), and metabolic enzymes such as cytochrome P450 and UDP-glucuronosyl transferases [5]—were responsible for biotransformation reactions, including glucuronidation and peptide/lipid hydrolysis [6]. The resulting highly integrated tubular transport–metabolic system yielded an oxygen consumption rate that was exceeded only by that of the myocardium and the cerebral cortex [7]. Consequently, the kidney functioned not merely as a passive filtration barrier but as an active organ that regulated intrarenal drug concentrations [8].

Because of the cortex’s high capacity for transporter-mediated proximal tubular secretion, drug metabolism within the kidney, biodistribution, and toxicity assessment are important factors influencing drug development [9]. For example, aminoglycoside antibiotics induced lysosomal rupture and oxidative stress in proximal tubule cells through the mitogen-activated protein kinase/c-Jun N-terminal kinase signaling pathway[10]; thiazide diuretics provoked acute kidney injury via effects on the renin–angiotensin–aldosterone system and tubular ion-transport pathways [11]; platinum-based chemotherapeutics caused direct cellular damage through the p53-mediated DNA damage response [12,13]; and certain nanomedicines damaged podocytes and induced foot-process effacement, thereby impairing glomerular filtration [14]. These widely prescribed agents pose severe nephrotoxic risks, necessitating rigorous evaluation of renal distribution and toxicity in preclinical development. We employed appropriate research models to indirectly assess these critical parameters [15].

Animal and cell models had been indispensable for studying renal drug handling, but inherent limitations remained. Animal kidney models exhibited compromised translational fidelity due to marked interspecies differences in the abundance and segmental distribution of key cortical transporters [16]. Microfluidic nephron units and other cell-based platforms lacked the complete renal metabolic microenvironment and therefore exhibited heterogeneous drug-metabolic behavior [17]. To address these gaps, ex vivo human kidney perfusion models were developed and progressively refined [18]. Nevertheless, the steadily growing population of patients with end-stage kidney disease limited the availability of healthy kidneys for use as research models [19]. Accordingly, discarded tumor-bearing kidneys (TKs) from renal cancer patients undergoing radical nephrectomy are available as samples. They are expected to serve as an important source for kidney models [20].

TK presented several practical advantages: (a) ischemic exposure was controllable because the surgeon could vary renal arterial clamping time intraoperatively [21]; (b) although kidneys exhibited high oxygen demand, organ function could be maintained for up to 24 h under hypothermic conditions [12]; (c) many TK specimens contained both residual normal parenchyma and malignant tissue, enabling comparative assessment of inter-tissue drug distribution [22]; and (d) renal cell carcinoma accounted for approximately 2% to 3% of all cancers, providing a substantial repository of pathological specimens [23]. These features suggested that TK was a promising resource for ex vivo renal modeling [24]. However, several challenges remained: (a) definition of inclusion criteria for specimen selection, (b) standardization of perfusion parameters, (c) objective evaluation of perfusion efficacy, (d) characterization of ex vivo renal function, and (e) quantification of modeling differences relative to conventional animal kidneys [25].

The present study addressed specimen screening, optimization of perfusion conditions, functional characterization, and comparative evaluation against traditional models [26] to establish a standardized ex vivo perfusion workflow for TK. A simple, reproducible, and spatially undemanding perfusion system was employed to achieve effective organ perfusion [27]. Ex vivo tumor-bearing kidney (ETK) maintained robust structural integrity and filtration function while being perfused with hypothermic (4 to 10 °C) saline. Kidneys with adequate perfusion produced a stable and slow urine output. Regarding model performance, the simple composition of saline allowed the ETK to display drug-induced injury in a more direct and interpretable manner. After cisplatin (CIS), gentamicin (GEN), or cephalosporins were introduced into the perfusate, the ETK exhibited drug-specific injury patterns across multiple dimensions, resembling clinical observations [28]. When indocyanine green (ICG) was added to the perfusate, the ETK showed a distinct drug-distribution profile. ICG accumulated primarily within normal renal parenchyma in the ETK, whereas it concentrated in tumor regions in the mouse orthotopic renal tumor model [29]. These findings further supported that species differences between mouse kidneys and ETK led to heterogeneous drug-distribution behaviors. Taken together, and compared to conventional models, the ETK perfusion platform offers 2 key advantages: (a) Overcoming limitations of animal models: Unlike animal models, ETK utilizes human kidneys to accurately recapitulate human-specific drug metabolism and distribution; (b) Whole-organ functional preservation: Unlike organoids or slices, ETK maintains whole-organ perfusion and filtration capacity for stable urine production and multidimensional injury assessment. Collectively, a high-fidelity human ETK perfusion platform was constructed and validated, providing a robust experimental basis for studies of renal drug metabolism and distribution, precision tumor imaging, and optimization of renal drug-delivery strategies [30,31].

## Results

### Establishing an extracorporeal circulation protocol for ETK

**Fig. 1.**
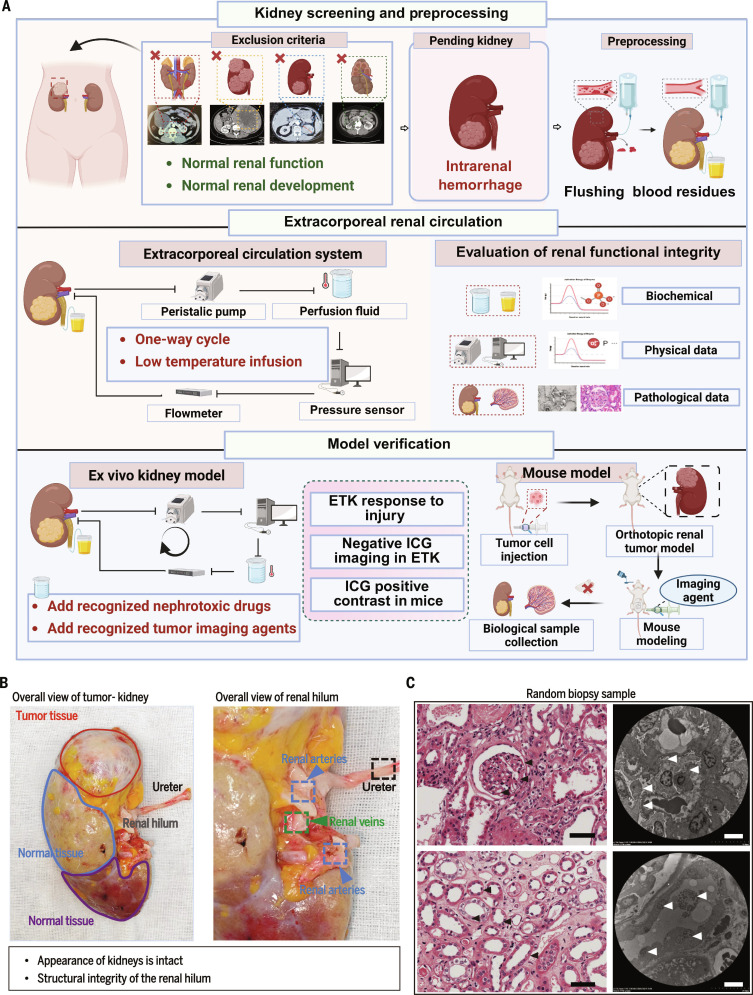
Flowchart and anatomical basis of the ex vivo tumor-bearing kidney (ETK) circulation system. (A) The workflow shows that clinical pathological samples were first screened and preprocessed. Perfusion conditions were then optimized in the ex vivo circulation system according to ETK physicochemical parameters. The performance of the ETK model was subsequently assessed, and indocyanine green (ICG) imaging in ETK and mouse models was compared to examine distribution differences between the 2 models. (B) Tumor-bearing kidney with intact renal hilum, including renal artery, renal vein, and ureter; the entire renal system remains closed. (C) Hematoxylin and eosin staining and electron microscopy indicated intact glomerular and tubular structures (black arrowheads); podocyte foot processes were relatively intact, with partial loss of the tubular brush border (white arrowheads). Black scale bar, 50 μm; white scale bar, 5 μm.

A complete extracorporeal perfusion platform facilitated the conduct of subsequent experiments by maintaining the functional integrity of ETK. To mitigate the heterogeneity among clinical TK specimens, initial specimen screening was performed; the inclusion criteria required that each TK not only permit an effectively closed perfusion circuit but also contain an appropriate proportion of tumor and adjacent normal parenchyma [32]. Based on these criteria, kidneys with congenital renal malformations, established renal insufficiency, or gross circulatory compromise were excluded to ensure the viability and sealability of the ETK circuit (Fig. 1A and Tables 1 and 2).

**Table 1.** Inclusion criteria for ETK

Inclusion criteria
Kidney and vascular system essentially normal, main artery diameter ≥ 0.4 cm, no suture-resistant arterial damage
No history of chronic kidney disease, preoperative renal function tests normal
≥3 clinicians independently evaluated normal renal tissue proportion ≥30% in the affected kidney
Minor lacerations manageable with surgical sutures

**Table 2.** Exclusion criteria for ETK

Exclusion criteria
Multiple branches supplying target area; vessel diameter <0.4 cm
ETK with congenital anomalies or previously diagnosed renal insufficiency
ETK tumors occupying >70%, normal tissue proportion <30%
Renal laceration during surgery not amenable to effective surgical closure

Following selection, each kidney underwent standardized preparation: (a) removal of perirenal adipose tissue; (b) exposure of the renal hilum; (c) systematic ligation and trimming of arterial branches; and (d) mobilization of the ureter, thereby establishing unambiguous surgical anatomy for cannulation [33] (Fig. 1B). Flushing step emphasized as critical: thorough irrigation reduced thrombus formation, preserved patency of the extracorporeal circuit, and minimized confounding effects on subsequent assays. Perfusion hardware was configured according to a principle of simplicity and robustness: a constant-flow peristaltic pump supplied stable primary circulation, and a reservoir sustained perfusate volume [34]. Pressure transducers and flow meters were incorporated to provide continuous hemodynamic readouts and auxiliary validation of circuit performance [35] (Fig. 1A).

Temperature control implemented to modulate metabolic demand: previous studies indicated that biochemical reaction rates approximately halve with each 10 °C decrement; accordingly, hypothermic preservation at 4 to 10 °C reduced renal oxygen consumption to roughly 10% to 20% of normothermic levels, thereby enhancing ETK tolerance to ischemia and extending the experimental window [36]. A cold workbench and a sterile ice box with high heat capacity were used to maintain target temperatures. During perfusion, timed sampling of perfusate and urine specimens enabled quantitative assessment of ETK injury markers and informed iterative optimization of perfusion parameters [37] (Fig. 1A).

Macroscopic and histologic assessments were used to evaluate organ integrity after perfusion. Coloration and tissue turgor were recorded, and random biopsies demonstrated preserved glomerular and tubular architecture with intact podocyte foot processes and well-defined cellular outlines, supporting the conclusion that the ETK microstructure remained intact under the standardized perfusion protocol [35] (Fig. 1B and C). Thereafter, the ETK model was interrogated for its responsiveness to nephrotoxic insults: nephrotoxic agents and clinically used imaging tracers were introduced via the circuit to evaluate both the model’s susceptibility to drug-induced injury and potential differences in intrarenal distribution relative to established animal models [38].

### Perfusion conditions for ETK

**Fig. 2.**
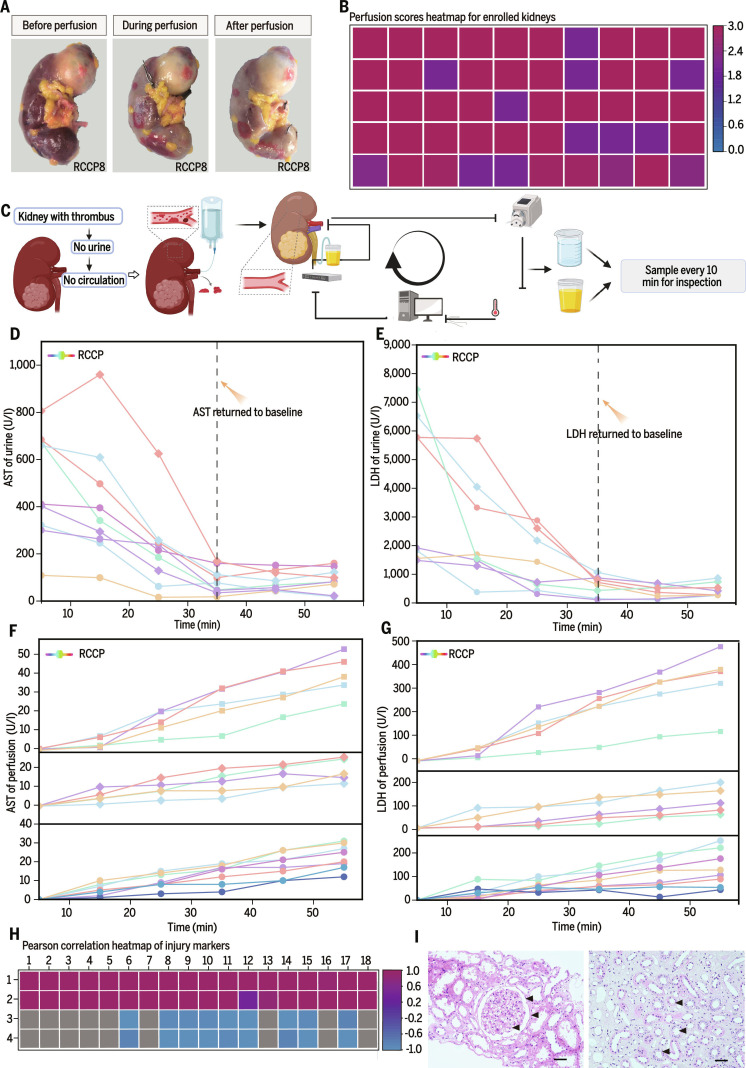
Optimization of ETK ex vivo circulation and pretreatment. (A) Sequential images of the ETK during preflushing display morphological changes caused by continuous perfusion. (B) The perfusion score heatmap shows that all 50 kidneys included in the study exhibited good perfusion, making them suitable for subsequent experiments. (C) Schematic of pretreatment shows that most residual blood in the ETK was cleared after completion of the pretreatment process. The ETK was then connected to the perfusion system, with urine and perfusate samples collected every 10 min for subsequent experiments. (D and E) Line graph showing changes in aspartate aminotransferase (AST) and lactate dehydrogenase (LDH) concentrations in ETK urine after the start of perfusion. (F and G) Line graph showing changes in AST and LDH concentrations in ETK perfusate after the start of perfusion. (H) Pearson correlation heatmap showing that AST and LDH in urine are negatively correlated with time within the first 30 min of perfusion, whereas they remain positively correlated in the perfusate. (I) ETK biopsy pathology confirms well-preserved microstructure (black arrowheads). Black scale bar, 50 μm

During perfusion, ETK did not always achieve effective reperfusion because true reperfusion injury was absent in this setting. In the preflush phase, the ETK underwent visible morphological changes as residual intrarenal blood was cleared (Fig. 2A); regions that were successfully filled became firm upon entry of the perfusate and subsequently produced a measurable amount of urine [39]. These changes were used to define a perfusion-quality scoring system that enabled quantitative assessment of reperfusion adequacy. ETK that achieved a perfusion score of ≥2 (Table 3), as determined by experienced surgeons, were considered suitable for downstream experimentation. A total of 50 TK met this criterion and constituted the overall sample for the study; these 50 ETKs were derived from 50 individual renal cell carcinoma patients (Fig. 2B and Figs. S1 and S2).

**Table 3.** Perfusion efficacy score

Perfusion quality score
Score 1: ETK showed no perfusion in the target region, soft texture, no obvious color change, no urine output, or urine output <0.5 ml at RBF = 70 ml/min
Score 2: ETK showed perfusion in the target region, firm texture on palpation, overall color lightened or target region lightened, slow and steady urine output; urine output ranged 1.0–1.5 ml/min at RBF = 70 ml/min
Score 3: ETK exhibited overall perfusion, firm texture on palpation, overall color pale pink or with small red areas, with slow and steady urine output; urine output ranged 1.0–1.5 ml/min at RBF = 70 ml/min

Each ETK was subsequently connected to the ex vivo perfusion platform, and biochemical changes in urine and perfusate were monitored to optimize perfusion parameters (Fig. 2C). Normal saline, widely used and chemically inert, was selected as the initial perfusate to avoid confounding the biochemical composition of collected samples. Aspartate aminotransferase (AST) and lactate dehydrogenase (LDH), commonly used markers of cellular stress and injury, were measured as surrogate indicators of tubular epithelial integrity [40]. After initiation of ex vivo perfusion, urinary AST and LDH declined toward baseline within approximately 35 min, whereas AST and LDH concentrations in the perfusate progressively increased over time (Fig. 2D to G). Pearson correlation analysis confirmed a negative association between urinary injury markers and perfusion duration within the first 35 min, and an obvious positive correlation between perfusate enzyme levels and time (Fig. 2H). Ultrasound-guided tissue biopsies performed at the end of the observation window showed preserved glomerular architecture, partial loss of the proximal tubular brush border, and overall intact cellular morphology with well-maintained tubular luminal contours (Fig. 2I).

### Urine output-guided optimization of the perfusion protocol

**Fig. 3.**
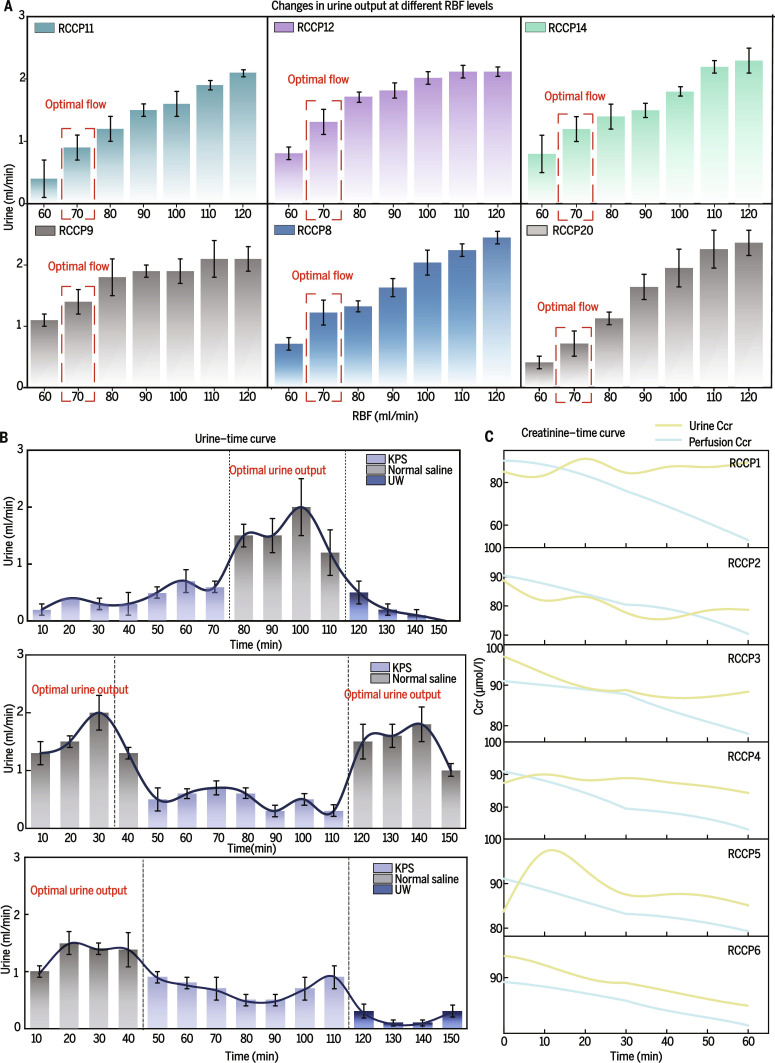
Perfusate type, flow rate, and ETK filtration characterization. (A) Urine output was positively correlated with perfusate flow rate, reaching approximately 1 ml/min physiological level when renal blood flow (RBF) reached 70 ml/min (*n* = 6). (B) The urine output line graph shows maximal production with saline perfusion (*n* = 3). (C) Perfusate and creatinine curves display sustained creatinine excretion in urine (*n* = 6).

Urine production capacity was a key functional indicator of the ETK, and an appropriate urine output served as an essential reference for establishing physiologically relevant perfusion conditions. To optimize perfusion parameters based on urine output, 6 ETKs were evaluated under different renal blood flow (RBF) rates. Urine output increased continuously within a defined range as RBF increased. When RBF reached 100 to 120 ml/min, this trend stabilized in a subset of kidneys. Notably, the most informative observation window occurred when RBF was maintained at 60 to 80 ml/min (Fig. 3A and Fig. S3C). Within this window, although inter-organ heterogeneity persisted, urine output demonstrated a steady and uniform upward trajectory. When the perfusate flow rate reached 70 ml/min, the ETK reproduced a urine output of approximately 1 to 1.5 ml/min, approaching the physiological rate (Fig. 3A). RBF corresponding to physiological urine production prevented mechanical injury to the glomerular microarchitecture induced by excessive flow; therefore, 70 ml/min represented the most appropriate RBF when normal saline was used as the perfusate (Fig. S3E).

Although normal saline could be used for perfusion, comparison with commercial perfusion or preservation solutions remained necessary. RBF was therefore set at 70 ml/min, and ETKs were sequentially perfused with University of Wisconsin solution (UW), kidney preservation solution (KPS), and normal saline to generate time–urine output curves. Regardless of perfusion order, normal saline consistently yielded the greatest urine output, UW produced the least, and KPS resulted in intermediate output [41] (Fig. 3B and Fig. S3A to C). Furthermore, within the RBF range of 70 to 90 ml/min, only the normal saline group exhibited robust flow dependence. Although KPS produced more urine than UW, its urine output remained unstable even with an extended observation window. Based on the high priority of urine output for ETK-based modeling, UW, as a static preservation solution, could not support urine production. Collectively, normal saline represented the most suitable standard perfusate for ETK modeling (Fig. 3B).

Creatinine is a widely used indicator of renal filtration function. After creatinine was added to the perfusate, its concentration decreased steadily during the 60-min observation period, and creatinine became detectable in the urine. Although physiological saline lacked oxygen-carrying capacity and essential nutrients, and individual kidneys displayed some heterogeneity in time–concentration profiles, creatinine concentration in urine similarly exhibited a declining trajectory (Fig. 3C).

### ETK response to drug-induced nephrotoxicity

**Fig. 4.**
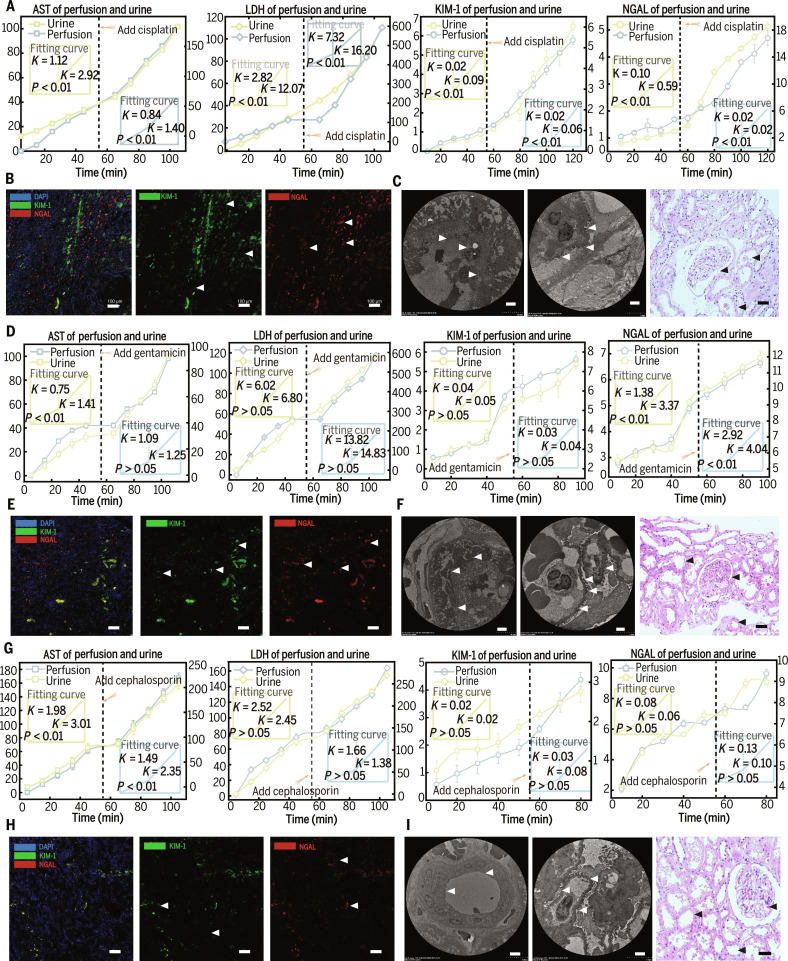
ETK response to drug-induced injury. (*n* = 6 per group). (A) Cisplatin administration in the perfusate; the fitted slopes of AST, LDH, kidney injury molecule-1 (KIM-1), and neutrophil gelatinase-associated lipocalin (NGAL) markedly changed. (B) Multiplex immunofluorescence (mIF) confirmed widespread expression of KIM-1 and NGAL (white arrowheads). (C) At the microscopic pathological level, tubular cell contours were disrupted, podocyte foot processes showed effacement and fusion (white arrowheads), glomerular structure was lost, and tubular epithelial cells were severely damaged (black arrowheads). (D) Gentamicin induced moderate drug-induced kidney injury, with changes in the fitted curves of injury markers weaker than those observed with cisplatin. (E) Partial tissue infiltration of KIM-1 and NGAL was observed. (F) Tubular epithelial damage, though cell contours remained visible. Some podocyte foot process fusion occurred, and glomerular structure was preserved. (G to I) Cep perfusion induced minimal ETK injury (G), with limited infiltration of injury markers (H) and preserved glomerular microstructure (I). White scale bar, 100 μm (mIF) and 5 μm (EM). Black scale bar, 50 μm.

ETKs were evaluated for their responsiveness to drug-induced injury as a measure of modeling efficacy. Following a 55-min stabilization period, nephrotoxic agents prepared in sterile normal saline were administered as a single bolus to achieve target concentrations of CIS (15 mg/l), GEN (100 mg/l), or cephalosporin (500 mg/l). This was followed by 50 to 60 min of continued perfusion to observe acute toxicological responses. CIS accumulated in proximal tubules via OCTs and induced intrastrand DNA crosslinks, activating p53, γ-H2AX, reactive oxygen species, and TLR4/NF-κB signaling pathways [42]. Following CIS perfusion, AST, LDH, kidney injury molecule-1 (KIM-1), and neutrophil gelatinase-associated lipocalin (NGAL) in urine and perfusate increased markedly. AST and LDH elevations indicated cellular stress, while KIM-1 and NGAL confirmed proximal and distal tubular injury [43]. Multiplex immunofluorescence revealed extensive KIM-1 and NGAL infiltration, and electron microscopy showed severe tubular damage, including brush border loss, epithelial cell structural disruption, nuclear fragmentation, podocyte foot process effacement, and near-complete obliteration of tubular lumens. The sections displayed tubular cell debris, focal epithelial loss, and glomerular disruption (Fig. 4A to C and Fig. S5A).

GEN induced nephrotoxicity, but weaker than CIS [44]. GEN uptake via cubilin triggered lysosomal rupture and subsequent molecular cascades [45]. AST and NGAL slopes in perfusate and urine were elevated, with NGAL showing greater sensitivity. Immunofluorescence confirmed broader NGAL infiltration and partial KIM-1 positivity. Electron microscopy revealed partially preserved brush borders, mild tubular epithelial vacuolization, largely intact tubular lumens, and focal foot process fusion, with glomerular filtration architecture maintained. Hematoxylin and eosin (H&E) sections showed mostly intact tubules and glomeruli (Fig. 4D to F and Fig. S5B).

Cephalosporins exhibited minimal acute nephrotoxicity within the perfusion window. Only AST showed minor changes; KIM-1 and NGAL remained largely unchanged. Immunofluorescence revealed minimal interstitial infiltration, slight tubular edema, and largely preserved tubular and podocyte architecture. H&E sections demonstrated intact tubules and glomeruli (Fig. 4G to I and Fig. S5C). To rule out artifacts from the perfusion system, we evaluated a saline-perfused control group. This group showed fully preserved structural integrity and baseline biomarker levels (Fig. S4). These observations indicated that ETK established on the ex vivo perfusion platform effectively responded to drug-induced injury, consistent with clinical pharmacological profiles of the tested agents.

### Imaging differences of ICG between ETK and orthotopic mouse renal cancer models

**Fig. 5.**
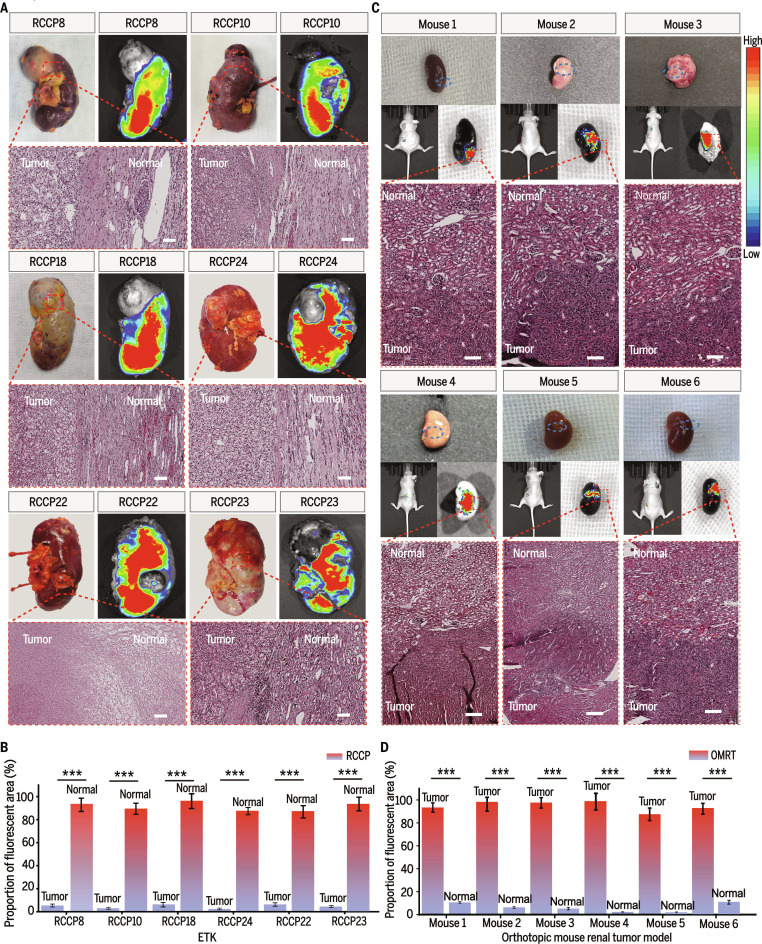
Imaging of ICG in ETK and mouse orthotopic renal tumors. (A) ICG preferentially accumulated in normal tissues within the ETK. (B) Fluorescence quantification analysis demonstrated that ICG accumulated more prominently in normal tissues. (C and D) ICG accumulated within tumor tissues in the mouse renal cancer model, and fluorescence quantification revealed a stronger signal intensity in tumor regions. White scale bar, 100 μm

ICG, an agent approved by the Food and Drug Administration for clinical imaging, provided a safe and direct means to visualize drug distribution in ETK and to compare it with distribution in mouse orthotopic renal cancer models [46]. In the 6 ETK samples included in the study, all kidneys exhibited negative ICG contrast during perfusion. The agent accumulated predominantly in normal renal tissue surrounding the tumor, with a clearly delineated tumor boundary [47]. This effect was consistent regardless of tumor location or growth pattern; both exophytic and endophytic tumors demonstrated distinct demarcation between tumor and adjacent normal tissue. Sampling at the tumor–normal interface confirmed that tissue morphology corresponded to the pathological diagnosis (Fig. 5A and B).

In contrast, in the mouse orthotopic renal cancer models, intravenous administration of ICG resulted in positive tumor contrast [48]. ICG preferentially accumulated within tumor tissue, with minimal enrichment in normal renal tissue. All 6 mouse models displayed consistent ICG distribution, and histopathological assessment confirmed that areas of contrast corresponded to tumor morphology (Fig. 5C and D). These findings indicated that ETK exhibited unique drug distribution characteristics compared with conventional animal models, highlighting its potential as a complementary platform for studying renal drug metabolism and intratumoral delivery.

## Discussion

In this study, we established and validated an innovative ETK perfusion model that demonstrated notable advantages in structural preservation, physiological function, toxicological response, and drug distribution [49]. This platform bridges the translational gap between conventional animal and cell-based models [50], advancing studies of human renal pharmacokinetics and toxicology, while highlighting the novel utility of TK obtained from clinical radical nephrectomy specimens [22].

The ETK model maintained robust macroscopic and microscopic integrity. Postperfusion tissue color, turgor, glomerular and tubular morphology, and podocyte foot processes observed via electron microscopy indicated that renal microarchitecture was largely preserved under ex vivo circulation [51,52]. These findings complement previous organ preservation and transplantation studies, which maintained tissue viability through hypothermic storage or perfusion [53]. By repurposing TK that would otherwise have been discarded, ETK represents a novel utilization of human renal tissue, transforming it into a quantifiable and reproducible experimental tool [54].

Inclusion criteria during kidney screening were tailored to ensure adequate characterization of ex vivo functional parameters [55]. While preoperative physiological assessment was particularly relevant for this study, the criteria can be adjusted for studies focusing on chronic kidney disease [56]. However, intact renal hilum anatomy and a closed-loop perfusion circuit remained essential [57]. Given the presence of variant arterial branches supplying distinct renal regions, perfusion adequacy in target areas and surrounding tissue was prioritized, with distal microvascular territories included at the discretion of experimental requirements [58].

During initial perfusion, urinary AST and LDH gradually declined to baseline within ~30 min [59]. This likely reflected warm ischemia during surgical arterial clamping and the accumulation of injury markers before ex vivo reperfusion [54]. Urine output stabilized within this timeframe, while perfusate mainly reflected ongoing stress from nutrient deprivation [52]. Prioritizing urine output during protocol optimization was critical, as it not only reflected physiological renal function but also provided essential biological samples for downstream studies [53]. The use of a constant-flow peristaltic pump and normal saline minimized mechanical injury and experimental interference [60]. Although nutrient-enriched or oxygen-carrying perfusates could extend ETK functional time, increased viscosity may hinder urine production [61]. For example, the UW solution, containing high-molecular-weight impermeants such as lactobionic acid, impeded urine output, consistent with its use as a static preservation solution [62]. In contrast, KPS, with mannitol as an osmotic stabilizer, allowed for relatively higher urine production (Fig. S3D).

The ETK model’s responses to nephrotoxic agents recapitulated known clinical and in vivo mechanisms. CIS perfusion markedly elevated AST, LDH, and kidney injury markers (KIM-1 and NGAL), with immunofluorescence and electron microscopy revealing severe proximal tubular damage and structural disruption [63]. GEN induced milder but detectable injury, aligned with cubilin-mediated lysosomal disruption [64]. Cephalosporins elicited minimal acute injury. These results underscore the ETK’s potential as a clinically relevant platform for drug safety evaluation [65].

ICG fluorescence revealed distinct drug distribution patterns in ETK compared with mouse tumor models [66]. In ETK, ICG accumulated preferentially in normal renal parenchyma, clearly delineating tumor boundaries, whereas it concentrated primarily within tumor tissue in mice [67]. This discrepancy is likely due to the fact that the accumulation of ICG is governed by the coordinated effects of hemodynamic perfusion and specific active transport mechanisms in renal tubular epithelial cells. Specifically, the transcellular transport of ICG—an anionic dye—relies heavily on basolateral OATs (such as OAT1 and OAT3) [68] for cellular uptake, and apical efflux pumps (such as MRP2 and MRP4) [69] for tubular secretion. Imaging results from both animal models and the ETK model demonstrated that marked interspecies differences in the abundance, spatial distribution, and substrate affinity of these key cortical transporters between human and mouse kidneys fundamentally alter ICG distribution patterns [70]. Ultimately, these factors may differentially affect drug distribution between the tumor and adjacent normal parenchyma under standardized ex vivo conditions. This suggests that ETK may more faithfully reflect human renal transport dynamics, providing complementary and human-relevant insights for targeted drug delivery and tumor imaging [71].

Despite its promise, the ETK model presents certain objective limitations. First, renal cell carcinoma is characterized by substantial pathological heterogeneity. Coupled with patient-specific factors—such as underlying comorbidities and preoperative medication histories—these inherent factors inevitably cause objective differences in the initial physiological states of the organs before the experiments begin. Therefore, while the 50 cases evaluated in this study establish a solid foundation, subsequent large-scale, multicenter cohorts remain necessary to fully account for these complex clinical variables. Second, the perfusion duration is strictly limited by the objective requirements of clinical diagnosis. Prolonged ex vivo perfusion may cause tissue alterations, which could compromise the accuracy of the patient’s definitive pathological diagnosis. To rigorously prioritize the patient’s clinical interests, the ETK platform is currently restricted to short-term functional evaluation and acute toxicity assessment, precluding long-term metabolic or chronic injury studies.

In conclusion, repurposing postsurgical TK for ex vivo perfusion represents a transformative approach, converting clinical tissue into a high-fidelity experimental platform [72]. The ETK model demonstrated structural stability, functional adaptability, and realistic drug response and distribution [73], providing direct human-relevant data for studies of renal transport, metabolism, tumor-targeted imaging, and optimization of drug delivery strategies [74]. Future integration of optimized perfusates, normothermic perfusion, and combination with other models may further enhance its utility in drug development, personalized therapy, and precision renal oncology [75,76].

## Methods

### Study design and sample collection

This case–control study was conducted at Harbin Medical University Cancer Hospital between August 2022 and August 2025. During the study period, after obtaining informed consent, a total of 238 nephrectomy specimens were collected. Specimens were excluded based on one or more of the following nonmutually exclusive criteria: preexisting renal insufficiency (40.0%), less than 30% normal renal tissue (29.8%), intraoperative renal injury (3.4%), congenital vascular or renal anomalies (2.9%), and perfusion-limiting circulatory obstruction (2.9%). After applying these criteria and conducting a feasibility assessment for ETK perfusion, 50 kidneys successfully met the predefined perfusion-quality standards and were retained for downstream analyses (Fig. S1). The study protocol was approved by the Institutional Review Board of Harbin Medical University Cancer Hospital (KY2023-63) and conducted in accordance with the Declaration of Helsinki.

Patients were preoperatively diagnosed with renal masses via imaging, and samples unsuitable for the study based on medical history were excluded. Renal specimens were obtained following radical nephrectomy and immediately placed on ice for transport. Before perfusion, surface blood was flushed from the kidneys, and large adipose tissue and renal parenchyma were bluntly dissected to expose the anatomical layers of the renal artery, vein, and ureter. The kidney surface was inspected for any damage or lacerations, and the feasibility of effective closure by surgical suturing was assessed. A ureteral catheter was inserted to allow for the separate collection of urine. During ex vivo perfusion, tissue sampling was performed using a biopsy needle. Depending on experimental requirements, normal renal parenchyma or tumor tissue was collected. Samples were fixed in 4% paraformaldehyde and stored at 4 °C for subsequent analyses.

### ETK simplified extracorporeal circulation platform

The ETK ex vivo perfusion system consists primarily of a tumor-bearing kidney, a unidirectional peristaltic pump, a reservoir, a thermometer, a pressure gauge, and a flow meter. The entire system is maintained at a low temperature of 0 to 4 °C. The peristaltic pump provides the primary driving force for circulation, while the reservoir retains perfusate recirculating between the renal artery and vein. The pressure gauge enables monitoring of intrarenal perfusion pressure under specific conditions. Within this system, perfusate is continuously pumped in a unidirectional, constant-pressure, and constant-flow manner through the renal artery and vein, completing a closed circulation loop. Additional components, such as the flow meter and pressure gauge, can be incorporated as needed to monitor and adjust perfusion parameters.

### Administration of nephrotoxic agents

Following a 55-min stabilization period, nephrotoxic agents were introduced into the perfusion circuit. The drugs were prepared in normal saline and administered as a single bolus into the reservoir to achieve the following target concentrations: CIS (15 mg/l), GEN (100 mg/l), and cephalosporin (500 mg/l). After dosing, perfusion was maintained for an additional 50 to 60 min to simulate clinically relevant exposure windows. A total of 18 ETKs were allocated for these drug-toxicity evaluations, with 6 kidneys assigned to each treatment group (CIS, *n* = 6; GEN, *n* = 6; cephalosporin, *n* = 6).

### Biochemical assays and baseline establishment

Perfusate and urine samples were collected at predefined intervals throughout the perfusion process. During the initial pretreatment phase, ETKs underwent a 55-min stabilization period, with samples acquired every 10 min. The pretreatment baseline was established by calculating the mean values obtained across this 55-min window. To assess generalized cellular injury, AST and LDH levels were measured by the institutional clinical laboratory using an automated clinical chemistry analyzer following standard operating procedures (*n* = 6 kidneys); values are reported in units per liter. Furthermore, to evaluate specific tubular damage, human KIM-1 and NGAL concentrations were quantified using commercial ELISA kits (KIM-1: BYAbscience, Cat# BY-EH111279; NGAL: BYAbscience, Cat# BY-EH111771) strictly according to the manufacturer’s protocols (*n* = 6 kidneys). Data are expressed as the mean ± standard deviation (SD). Drug-induced responses are presented as both absolute measurements and relative changes from the baseline, as detailed in the respective figure legends.

### Establishment of orthotopic renal cancer xenograft model

All animal experiments complied with the Guide for the Care and Use of Laboratory Animals and were approved by the Institutional Review Board of Second Affiliate Hospital of Harbin Medical University (GJZDYF2024-001). Female BALB/c nude mice (6 to 8 weeks, 16 to 18 g) were anesthetized with tribromoethyl alcohol and placed in the left lateral decubitus position. Then, the skin and abdominal muscles of the mice were incised, followed by kidney extraction. Subsequently, Renca-Luc cells were implanted beneath the renal capsule. Tumor engraftment was confirmed via In Vivo Imaging System (IVIS) at 1 week postimplantation.

### In vivo and ex vivo imaging of an orthotopic renal cancer xenograft model

After confirming tumor growth in the kidney, mice (*n* = 6) received a single tail-vein injection of ICG (1 mg/kg). Then, whole body IVIS imaging (*λ*_ex_ = 745 nm, *λ*_em_ = 840 nm) was performed at 5, 10, 15, 30, 45, 60, 90, 120, and 180 min postinjection. At the study endpoint, mice were euthanized, and kidneys were harvested ex vivo for fluorescence quantification (radiant efficiency, p/s/cm^2^/sr/μW/cm^2^) and histopathological validation. No animals or data points were excluded from analysis.

### H&E staining

Renal tissue samples were fixed in 4% paraformaldehyde at 4 °C for 24 h and subsequently dehydrated through a graded ethanol series. Samples were embedded in paraffin and sectioned at 4 μm thickness. Sections were deparaffinized in xylene, rehydrated through descending ethanol concentrations, and rinsed in distilled water. The sections were then stained with hematoxylin for 5 to 10 min, rinsed in running tap water, and differentiated in 1% acid alcohol. Following bluing in 0.2% ammonia water, sections were stained with eosin for 2 to 5 min, dehydrated, cleared in xylene, and mounted with a coverslip. Stained sections were examined under a light microscope (e.g., Olympus BX53) for assessment of renal morphology, including glomerular and tubular structures.

### Quantitative imaging and fluorescence analysis

To objectively quantify fluorescence intensity, 3 nonoverlapping regions of interest (ROIs) of uniform size were randomly delineated within both the tumor parenchyma and the adjacent normal renal tissue for each specimen. To strictly prevent selection bias, ROI placement was not based on fluorescence intensity; rather, it was anatomically guided by corresponding bright-field images and retrospectively verified by H&E-stained sections [77]. Quantitative analysis was performed using ImageJ software (NIH, Bethesda, MD) [78]. To eliminate nonspecific tissue autofluorescence and ensure data fidelity, a uniform background correction was applied to all images using a rolling-ball subtraction algorithm prior to measurement. Furthermore, to minimize subjective error, all ROI delineations and subsequent mean fluorescence intensity (MFI) quantifications were independently performed by 2 blinded investigators who were unaware of the experimental grouping, with the averaged values utilized for final statistical analysis.

### Multiplex immunofluorescence staining

Renal tissue sections were dewaxed and rehydrated in an automatic instrument following this protocol: Xylene I (10 min) → Xylene II (10 min) → 100% ethanol I/II (5 min each) → 95%/85%/70% ethanol (5 min each) → double-distilled water (ddH_2_O, 5 min × 2). For antigen retrieval: 0.01 mol/l citrate buffer (pH 6.0) was boiled in a retrieval container; sections were submerged, incubated at 95 to 100 °C (gentle boil) for 15 min, cooled naturally to room temperature (RT, ~30 to 40 min) with the buffer, then washed 3× with 0.01 mol/l phosphate-buffered saline (PBS) (5 min each) to remove residual buffer. After blotting excess PBS, sections were covered with 5% bovine serum albumin blocking solution and incubated in a humidified chamber at 37 °C for 30 min (to block nonspecific binding). Primary antibodies against NGAL (abcam, ab125075) and KIM-1 (abcam, ab316854) (1:200 dilution in 1% blocking solution) were applied, and sections were incubated overnight at 4 °C. The next day, sections were rewarmed at RT for 30 min, washed 3× with 0.01 mol/l PBS (5 min each), then incubated for 1 h in the dark with Abcam Alexa Fluor 488 goat anti-rabbit and Alexa Fluor 594 goat anti-mouse secondary antibodies (1:500 dilution in 0.01 mol/l PBS). Unbound secondary antibodies were removed by 3× PBS washes (5 min each, dark). Nuclei were stained with 4′,6-diamidino-2-phenylindole (1:10,000 dilution) at RT for 8 min, followed by 2× quick PBS washes (2 min each). Excess moisture was blotted (avoiding tissue desiccation); 1 to 2 drops of anti-fluorescence quenching mounting medium were added, and sections were coverslipped (no air bubbles), sealed with neutral balsam, and air-dried at RT for ~10 min. Images were captured via fluorescence microscope and analyzed with ImageJ software.

### Electron microscopy sample preparation process

Precise sampling: After obtaining kidney tissue via surgery or biopsy, immediately cut it into tiny tissue blocks smaller than 1 mm^3^ (about the size of a grain of rice) with a sharp blade, avoiding compression damage. Rapidly fix the tissue with a specialized fixative (such as glutaraldehyde) to prevent cellular autolysis. Dehydration and embedding: Dehydrate the tissue blocks through a graded series of alcohols (from low to high concentration), then embed them in epoxy resin. Place the embedded blocks in an oven to cure, forming a hard “tissue resin block” in preparation for sectioning. Ultrathin sectioning: Using a diamond knife on an ultramicrotome, cut the embedded blocks into slices only 50 to 80 nm thick. Float the sections on water and then transfer them onto copper grids (special carriers for electron microscopy). Staining and contrast enhancement: Place the copper grids in staining solutions such as uranylacetate and lead citrate. The deposition of heavy metals creates contrast among cellular structures (e.g., basement membranes and mitochondria), facilitating observation under the electron microscope.

### Statistical analysis

All statistical analyses and data visualizations were performed using GraphPad Prism (version 9.0; GraphPad Software, Inc.) and R software. Continuous quantitative variables are expressed as the mean ± SD. Differences between 2 independent groups were assessed using an unpaired, 2-tailed Student *t* test. For the continuous monitoring data, piecewise linear regression (segmented regression) was employed to determine the phase-specific fitted slopes of injury markers, accurately reflecting their distinct release rates across different experimental intervals. The correlation between continuous variables, such as injury markers and perfusion time, was evaluated using Pearson correlation coefficients. Statistical significance was defined as a 2-sided *P* < 0.05, with levels of significance indicated as follows: *P < 0.05, **P < 0.01, and ***P < 0.001.

## Ethical Approval

The study protocol was approved by the Institutional Review Board of Harbin Medical University Cancer Hospital (Approval No. KY2023-63) and conducted in accordance with the Declaration of Helsinki. Written informed consent was obtained from all participants before sample collection.

## Data Availability

The data supporting the findings of this study were generated and analyzed during the current study. Because of ethical restrictions and the involvement of patient-derived specimens, the raw data were not publicly deposited. Processed data and materials relevant to the conclusions were included within the article and the Supplementary Materials. Additional data were available from the corresponding authors upon reasonable request and with appropriate institutional approval. Furthermore, the vector graphics involved in this article are derived from the BioRender public database. The usage rights for the public database were obtained through authorized channels, involving no infringement or piracy (https://app.biorender.com/).
