# Supplementary figures and images for: An Ex Vivo Patient-Derived Tumor-Bearing Human Kidney Model Recapitulates Drug Toxicity and Metabolic Distribution
Source: Research (Wash D C). 2026 May 21;9:1257. doi: 10.34133/research.1257 (PMC13191094; doi:10.34133/research.1257)

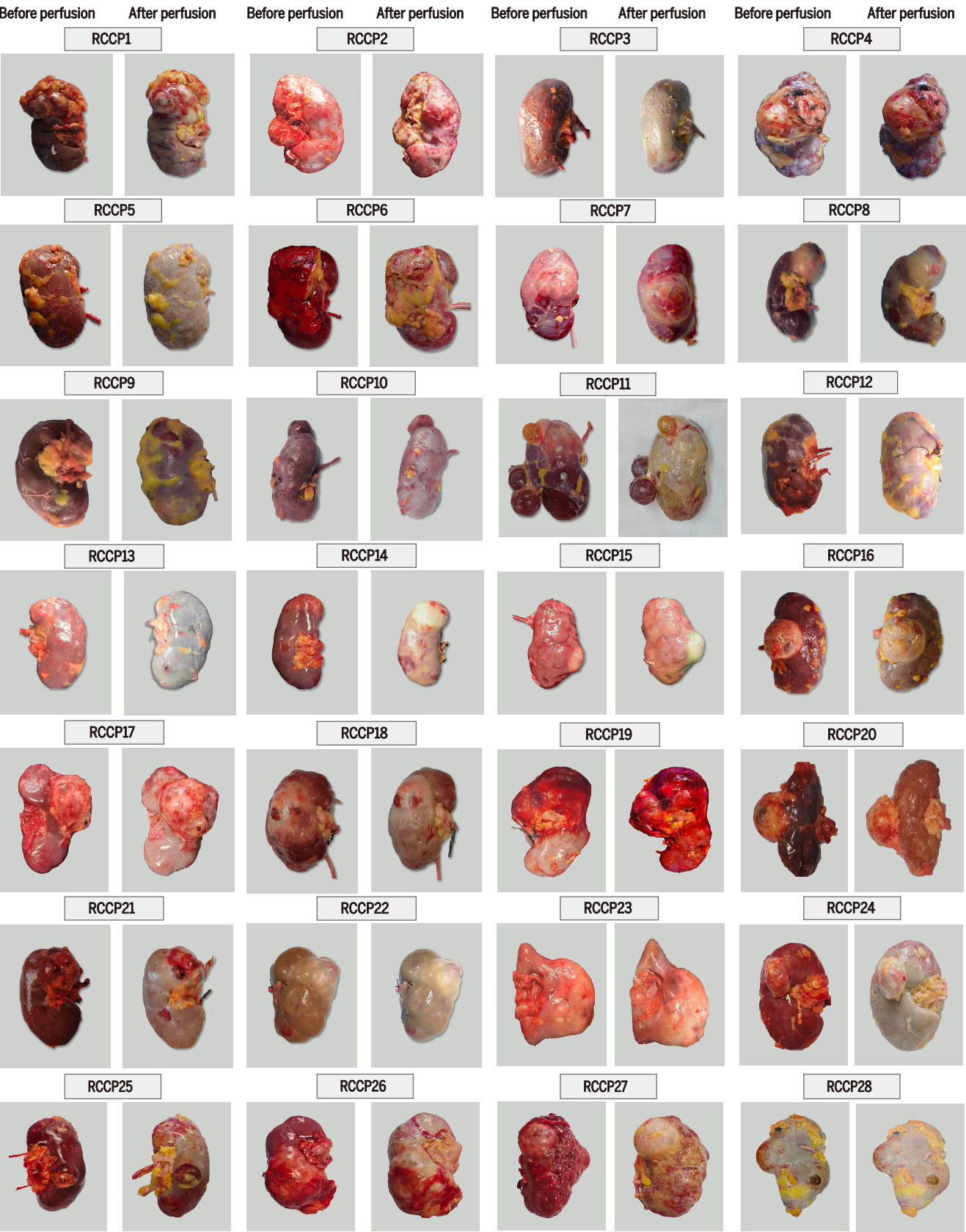

Supplement: Supplementary 1 — Figs. S1 to S5 Table S1 [file research.1257.f1.zip › FIG.S1.pdf]

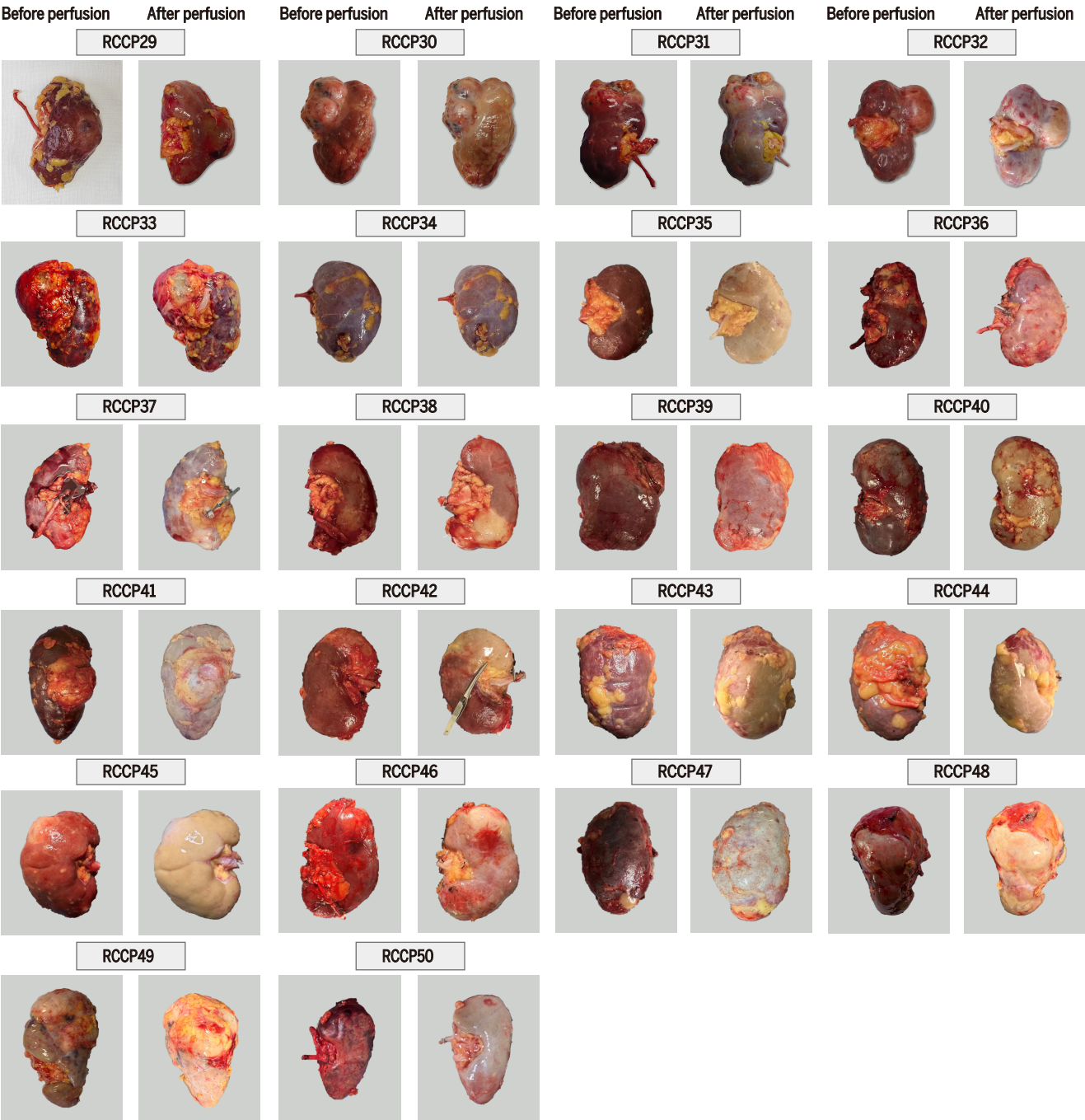

Supplement: Supplementary 1 — Figs. S1 to S5 Table S1 [file research.1257.f1.zip › Fig.s2.pdf]

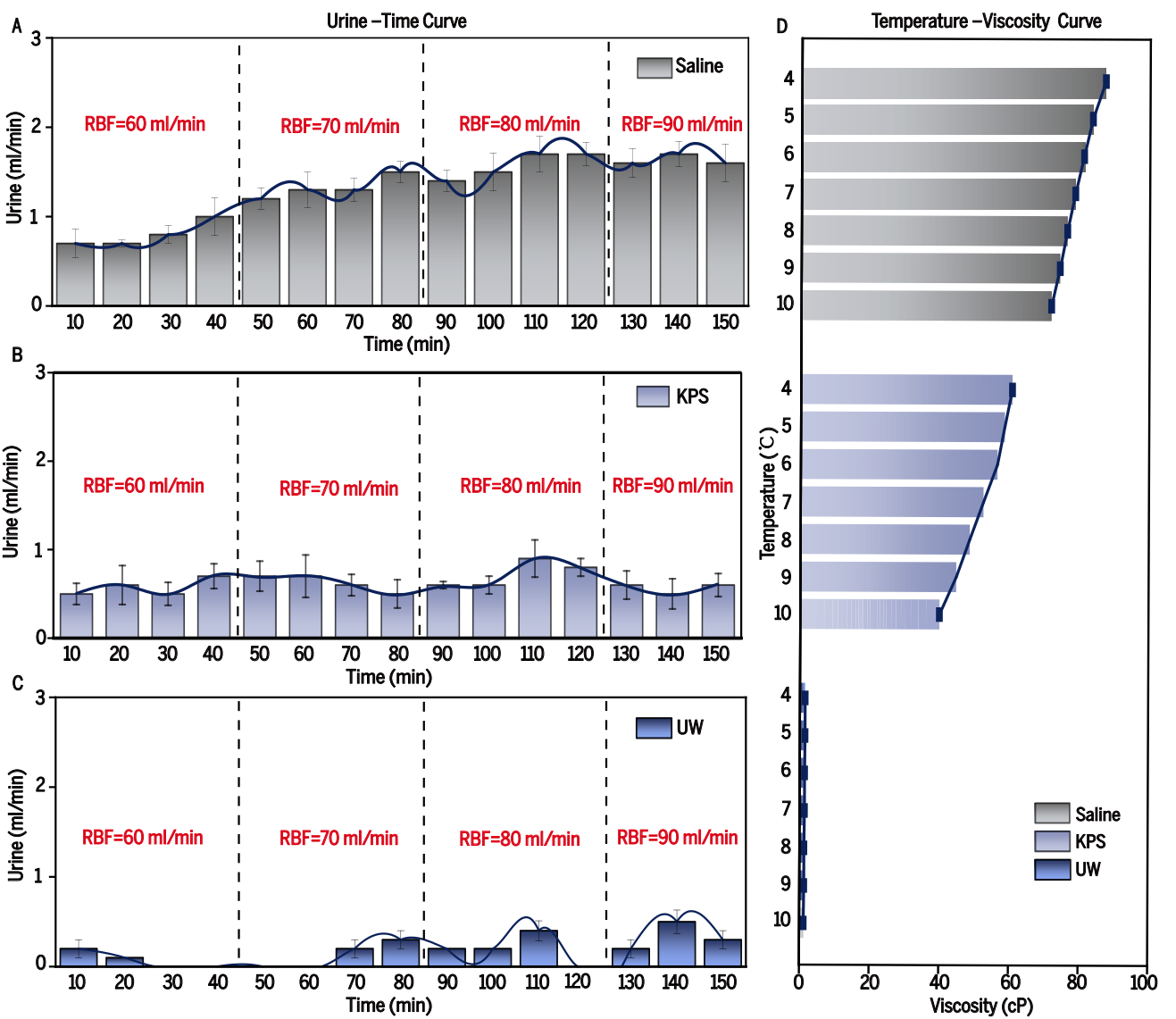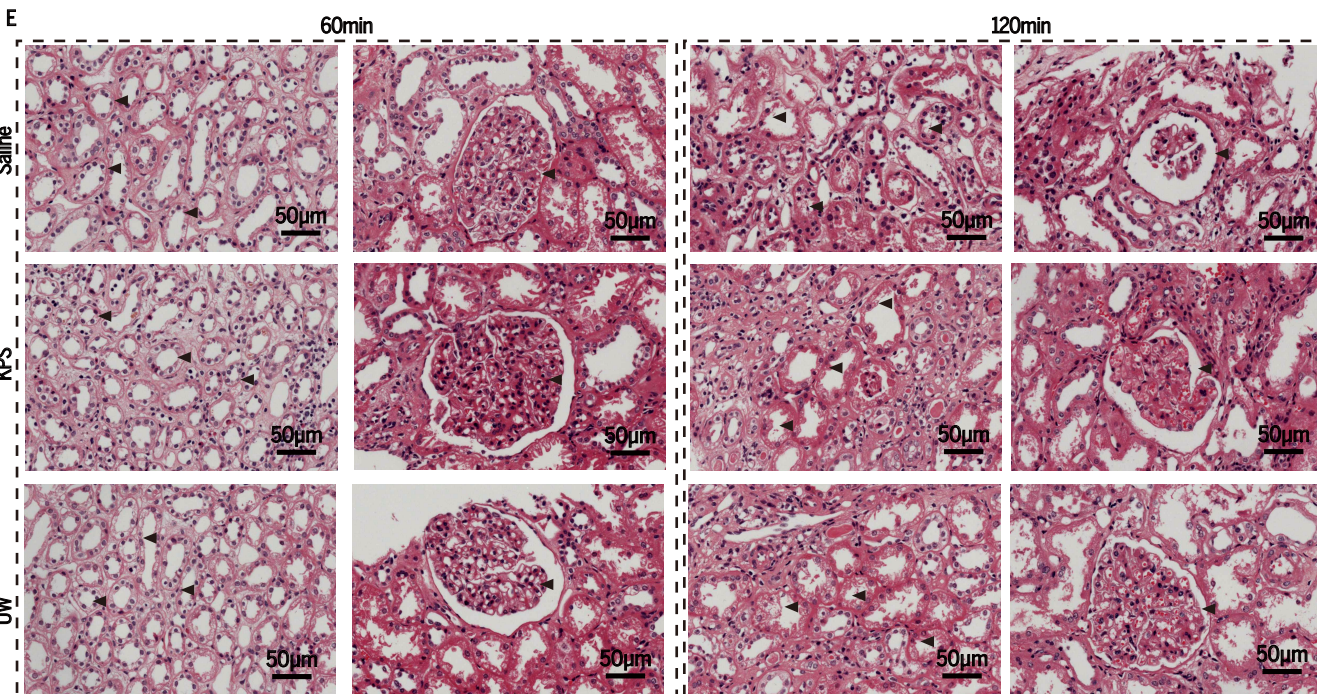

Supplement: Supplementary 1 — Figs. S1 to S5 Table S1 [file research.1257.f1.zip › FIG.s3.pdf]

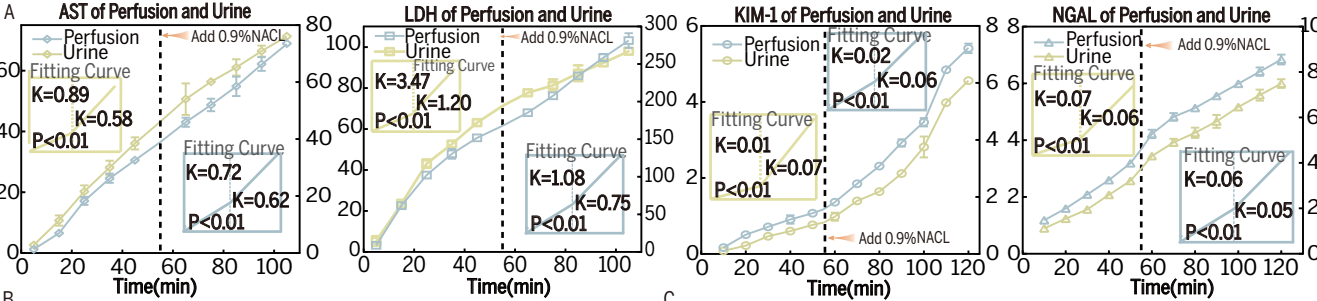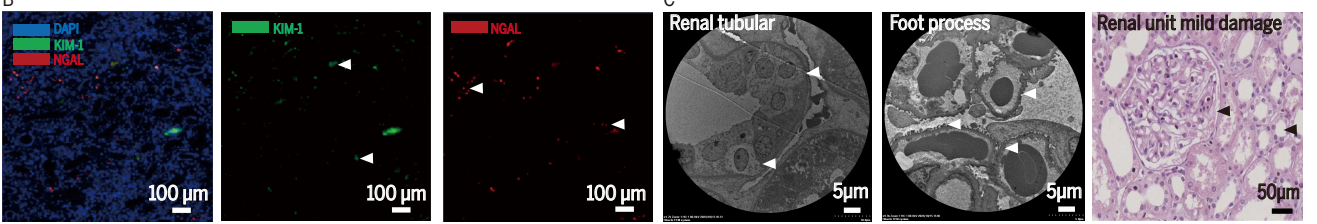

Supplement: Supplementary 1 — Figs. S1 to S5 Table S1 [file research.1257.f1.zip › fig.S4.pdf]

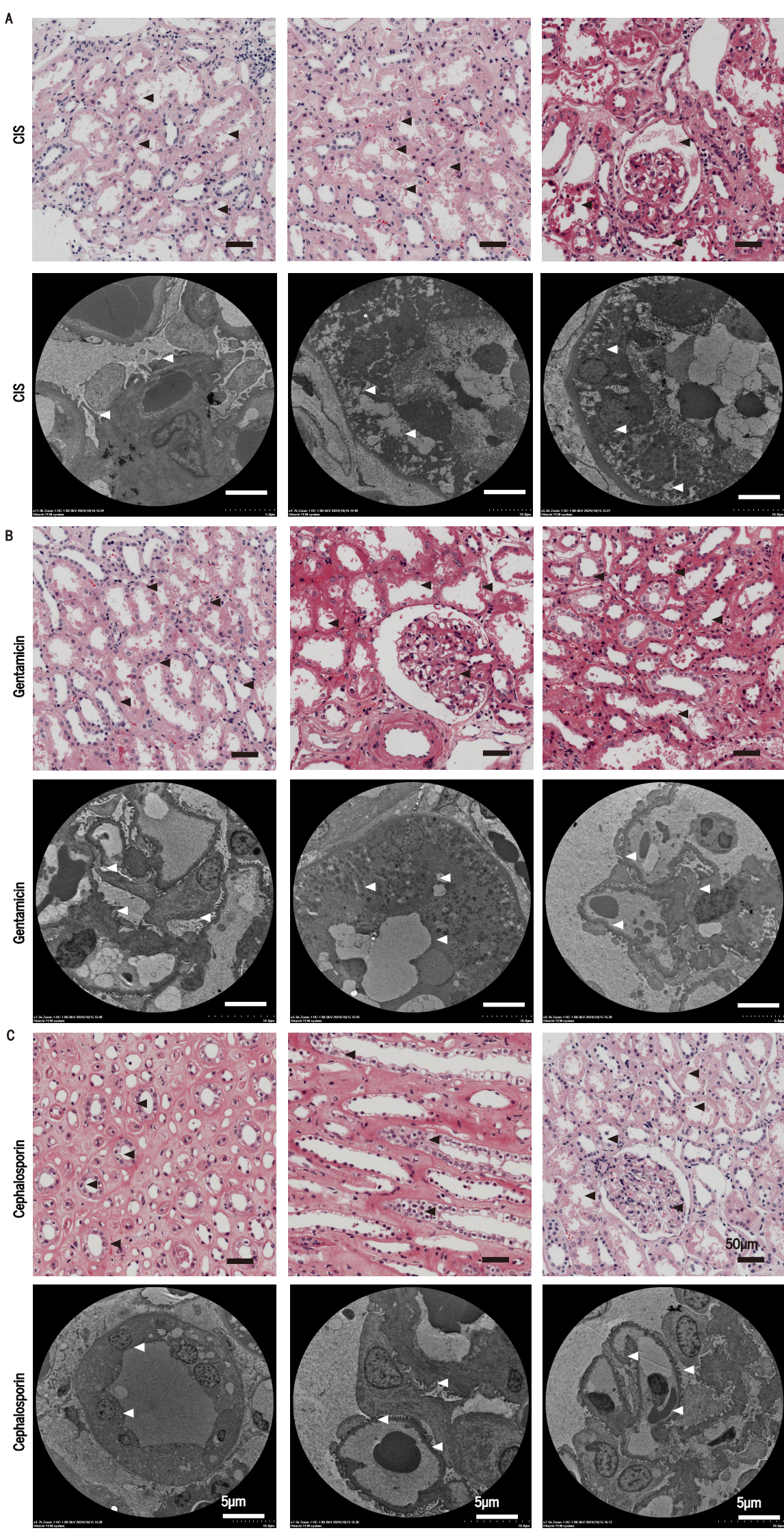

Supplement: Supplementary 1 — Figs. S1 to S5 Table S1 [file research.1257.f1.zip › Figs5.pdf]
